# Research on network security vulnerability risk contagion in software supply chain based on system dynamics

**DOI:** 10.1371/journal.pone.0335128

**Published:** 2025-11-21

**Authors:** Hui Cai, Qiang Xiong, Shuai Lian

**Affiliations:** 1 Sino-British College, University of Shanghai for Science and Technology, Shanghai, China; 2 School of Management, Jiangsu University, Zhenjiang, Jiangsu, China; Cardiff Metropolitan University - Llandaff Campus: Cardiff Metropolitan University, UNITED KINGDOM OF GREAT BRITAIN AND NORTHERN IRELAND

## Abstract

Software supply chains have emerged as a critical battleground in cyberspace security, with their compromise posing direct threats to critical infrastructure and information systems. The inherent multi-level structures and complex interdependencies among supply chain entities have introduced novel challenges in network and information security. This study investigates the contagion mechanisms of information security risks in software supply chains, aiming to identify key factors influencing risk propagation and evaluate effective defense strategies under multi-layer network conditions. We employ system dynamics (SD) modeling to construct a risk contagion framework for software supply chains, incorporating multi-layer network structures. Dynamic simulations are conducted to analyze risk transmission patterns under different attack and defense scenarios. The simulation results show that the risk transmission rate of software supply chain information security is influenced by the attack path. As compared to random attacks, selective attacks result in a faster risk transmission. In terms of defense strategy, increasing information security investment and improving the level of software quality are more effective for defense against random attacks. In terms of governance measures, increasing technological progress is more effective as compared to reducing the vulnerability rate. The results show that the marginal benefits of the technological progress rate show a decreasing trend. The study quantitatively validates the cascading effects of security risks in multi-layer supply chain networks and provides actionable insights and establishes a system dynamics foundation for predictive risk assessment in complex software supply chain ecosystems.

## Introduction

With the wide application of information technology, many individuals, firms, and organizations have employed software products to support their entertainment and commerce activities. Software security has become an area of significant concern for countries around the world, following traditional security domains such as homeland security [1], supply chain security [2], and food security [3,4]. The traditional attacks usually target the software vulnerabilities. However, in recent years, attackers have shifted their focus from software vulnerabilities towards software supply chains, i.e., the security space of software has extended from software to the boundary of code, modules, and services within the software [5]. Sonatype reports a 650% year-over-year increase in detected supply chain attacks (on top of a 430% increase in 2020) targeted toward upstream open source repositories. The U.S. government is so concerned by software supply chain security deficiencies that a whole section of Executive Order 14028, “Improving the Nation’s Cybersecurity,” issued 12 May 2021, is focused on new compliance requirements for government vendors to enhance supply chain security [6].

The R&D model is continuously evolving due to a ubiquitous demand for software. Traditionally, firms purchase software products from closed source firms, in which source code is closed and unavailable to users. This classical business model of software development is challenged by open source software, which gives users access to its source code and the freedom to use the software, improve it, fix its bugs, augment its functionality, and redistribute it for free [7]. According to the official website of CVE (Common Vulnerabilities & Exposures), there were a total of 5728 open-source vulnerabilities released in 2020, with a proportion of 8.83% super-high-risk vulnerabilities and 46.91% high-risk vulnerabilities. In 2021, the popular logging library log4j, used by more than 35,000 Java packages, allowed an attacker to perform remote code execution by exploiting an accidentally injected insecure Java Naming and Directory Interface lookup feature, which is enabled by default in many versions of the library. Both the SolarWinds and log4j events were driven by the software supply chain, whereby software products include “upstream” components as well as dependencies, which may be maliciously or accidentally vulnerable.

This has led to more complex information security issues in the software supply chain. It is noteworthy that the security issues at any stage of software design and development directly affect the security of all downstream processes in the supply chain, thus significantly expanding the scope and impact of attacks [8]. In addition, the features, such as significant fragmentation of software development, contribute considerably to the rapid spread of code pollution, eventually affecting the midstream and downstream users [9]. The well-known examples of software supply chain attacks include the SolarWinds supply chain attack [10], the Log4j vulnerability attack [11], and the GitHub authentication token attack [12].

Software is complex, not only due to the code within a given project, but also due to the vast ecosystem of dependencies and transitive dependencies upon which each project relies [8]. Vulnerabilities often require coordination between multiple parties [13]. Effective collaboration requires a clear understanding of contagion mechanisms through which information security risks propagate across enterprises within the software supply chain.

By constructing an information security risk contagion model for software supply chains under a multi-layer network structure, this study explores the microscopic mechanisms of risk contagion in software supply chains within the field of information security. It characterizes the heterogeneity of information security risk contagion in software supply chains under multi-layer network structures, and provides new insights for research on assessing the security compromises of software supply chains caused by information security risk contagion.

The remainder of this paper is organized as follows: First, a comprehensive literature review is presented. Then, research hypotheses are proposed, and a theoretical model using system dynamics methodology is constructed. Next, system dynamics simulation experiments are conducted and the results are analyzed. Finally, the concluding section discusses how these proposals might be applied and their expected consequences.

## Literature review

The software supply chain refers to a system in which software is written based on one or more levels of software design and development. This software is delivered from the software supplier to the user through software delivery channels [14], including third-party platform resources (e.g., open-source modules), software intermediate developers, final integrator and service providers, and other multiple participants. Currently, the repeated use of functional modules enhances the dependency between different software. This makes software supply chain network systems are not simple single-layer networks. Instead, these systems are multi-layered complex networks comprising software supplier network, software user network, and external information security environment of the supply chain, such as potential hackers [15,16]. Limiting to a single-layer network structure for studying the defects of single node and communication between nodes, it is more accurate to consider that each software supplier corresponds to a different layer in the multi-layer structure. This is the extension of the classical complex network and the hypergraph model, which lies between the multidimensional network and the interdependent network [17]. In addition, a derivation of the information security risk caused by network attack on a certain layer may lead to the cascading collapse of the entire software supply chain network [15,18]. The multi-layer complex network structures of software supply chains mainly include the following layers: potential hacker attack layer, software user layer and software supplier layer [19,20]. When a software supplier delivers a software product to an end user by using the internet as an intermediary or link, a public vulnerability in the network may lead to high-risk defects in the overall software supply chain [21]. Given the numerous entities involved in the multi-layer network of the software supply chain – along with the inherent complexity and inadequacy of its ecological relationships, the lack of complementary and mutually beneficial relationships [22], and the considerable heterogeneity of interests among entities – the poor handling or uncoordinated response to a security vulnerability can profoundly aggravate the contagion of information security risks throughout the entire network.

Recent studies in IoT and network security reveal increasing convergence of multidimensional technologies. Yin et al. [23] designed an anomaly detection framework combining deep auto-encoders with capsule graph convolution, optimized via Sparrow Search Algorithm for 6G heterogeneous environments. Concurrently, Waqas et al. [24] demonstrated machine learning-driven botnet detection in cloud-based IoT systems. Architecturally, Laghari et al. [25] established a blockchain-enabled distributed ledger (Lightweight-BIoV) for IoV trust management, while Ali et al. [26] proposed a confidentiality-aware cloud service model (C2aaS) to strengthen data governance. Notably, cross-domain integration emerges through Laghari et al. [27]‘s hybrid neural network for medical diagnostics and Yin et al. [28]’s attribute-based encryption for text privacy, collectively underscoring cybersecurity’s fusion with healthcare and cryptographic innovations.

Recent advances in computational optimization and adaptive algorithms demonstrate significant applicability in enhancing system security. In hardware architecture, Jafarzadeh et al. [29] introduced a buffering fault-tolerance mechanism for Network-on-Chip (NoC) systems, achieving a 43% reduction in packet loss under transient errors through dynamic virtual channel allocation. Addressing cybersecurity challenges, Alzubi et al. [30] developed a malware detection model integrating quantum mayfly optimization with encoder-decoder LSTM networks, which attained 96.3% classification accuracy while reducing computational overhead by 29%.

System dynamics (SD) is the science of studying complex information feedback systems. These systems systematically and dynamically analyze the research problems from qualitative and quantitative perspectives [31,32]. It is notable that system dynamics is flexible and universal enough for studying the complex mechanisms of single or multi-layer network threats. This provides a reference for exploring the mechanism of information security risk contagion in software supply chain. This work identifies the information security risk contagion factors of software supply chain by considering the multi-layer network based on system dynamics modeling. This work also presents a feasible method to prevent and control the information security contagion of software supply chain in multi-layer complex network.

## Description and analysis of the proposed model

### Model description

If a hacker discovers a vulnerability in a node in the software supply chain and launches an attack, the whole system may get affected by the spatial local collapse caused by the malicious attack on a single node due to the interdependence of nodes in the software supply chain. The local collapse may lead to a complete failure of the software supply chain network within a given radius of disturbance. In many cases, the local information security risks in interdependent spatial networks cause more damage to the multi-layer networks as compared to equivalent single-layer supply chain networks. If the local information security risk in software supply chain is higher than the critical value, it spreads and infects the entire software supply chain system within the scope of various levels of network systems.

**Fig 1:**
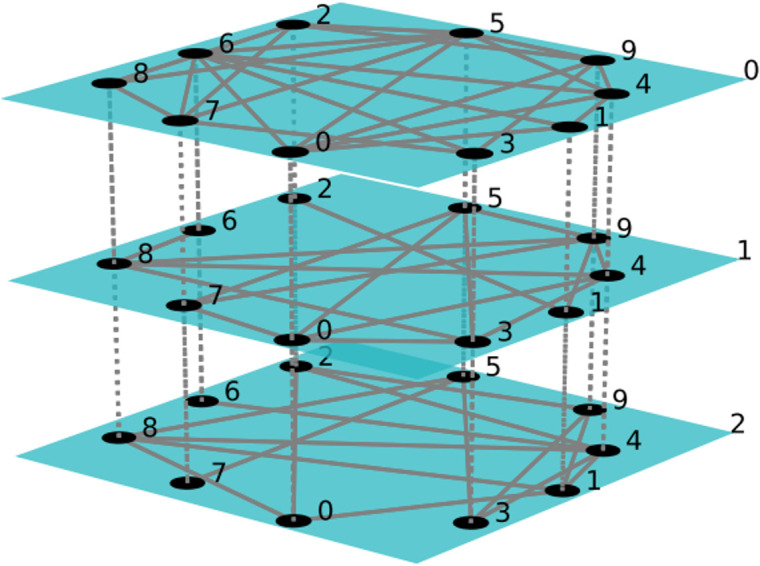
Multi-layer network diagram of information security risk contagion in software supply chain.

**Fig 2:**
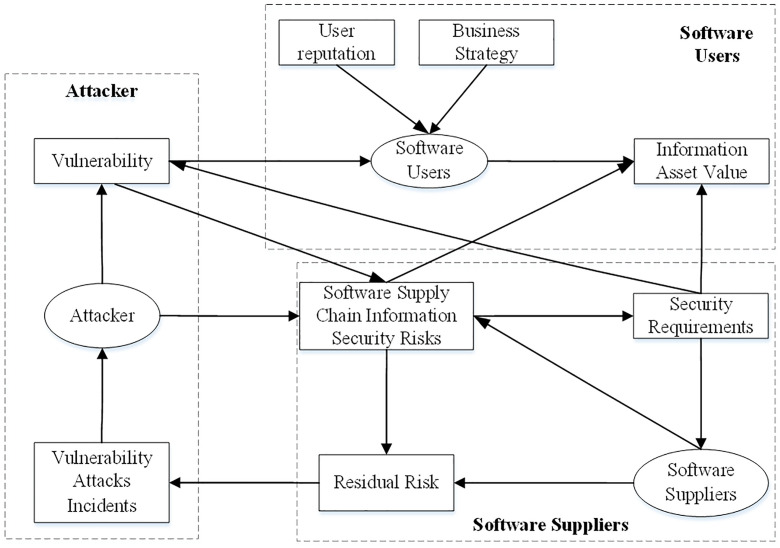
Causal relationship diagram of information security risk contagion in software supply chain.

Fig 1 describes the information security risk contagion in software supply chain by considering a multi-layer network with multiple physical nodes and vulnerability attack layers, software user layer, and software supplier layer, i.e., $B=\left(L_{B},Y_{B},Z,1\right)$, where, B represents the hierarchy, Z represents the node set in the supply chain, Y represents the edge set in the software supply chain, and 0,1,2 represent the software supplier layer, vulnerability attack layer, and software user layer, respectively. The nodes are connected to each other in pairs within and across the layers, with solid lines representing the inner edges of the layer, and the dotted and arc lines representing the edges between layers, taking into account: (1) links within different groups; (2) the nature of links and the relationship between elements belonging to different layers; (3) specific nodes belonging to each layer involved. This work divides the software supply chain into three layers, including vulnerability attacker, software supplier, and software user, thus forming three interacting subsystems. The specific composition is as Fig 2:

### Model assumptions

In order to clarify the boundaries of the software supply chain system, based on the reasonable information security assumptions in the process of adopting enterprise information security technology [33], a system dynamics model of network security vulnerability risk contagion in the software supply chain is constructed, and the following assumptions are made.

**Hypothesis 1:** The contagion of network security vulnerability risk in the software supply chain has internal and external characteristics, not only the attack of external hackers, but also the information security risk caused by the operation error of internal employees, this paper assumes that the external environment is uncertain, and the internal environment is relatively stable.

**Hypothesis 2:** The increased risk of software supply chain remnants can be exploited by hackers, further inducing new exploits.

**Hypothesis 3:** The costs of software vendors mainly include software development, patch development, and employee information security training.

**Hypothesis 4:** An increase in the level of hacking technology will accelerate the rate of malware infection.

**Hypothesis 5:** The improvement of information security technology can continuously improve the prevention and control effect of security governance measures.

**Hypothesis 6:** The rising risk of vulnerability attacks can prompt software vendors to accelerate the efficiency of patch development [33].

### Modeling of system dynamics

This paper divides the software supply chain into three levels: vulnerability attackers, software vendors, and software users. The relevant parameters at each level are described below:

(1) Software supplier layer: The software suppliers conduct initial software research and development according to the information security cooperation contract signed with each software user (information interaction and sharing between software suppliers and software users). Let the information sharing of software suppliers be $K_{s}$ and the sharing efficiency be $\gamma_{s}$. The initial safety factor of the software a is aimed at reducing the risk of cybersecurity vulnerabilities in the software supply chain by by implementing security governance measures through software research and development. In later stages, it relies on the technological progress for developing the vulnerability patches to minimize residual risks as much as possible. The other involved parameters include: the effectiveness coefficient of preventive measures d, rate of technical progress $t_{s}$, rate of technical decay $t_{a}$, the number of successfully managed information security risks u, rate of increase in vulnerability $V_{i}$, rate of the reduction in vulnerability $V_{r}$, and the total benefit E.
(2) Software user layer: Due to the drive of enterprise business strategy G and the information asset value A, the beneficial information M and reputation R of software users, certain information security risks increase. Based on the asset value and reputation value of software users e, these will prompt the software users to increase investment in information security I (including investment in security equipment $I_{s}$, investment in information security education $I_{e}$, etc.) and improve the level of information security management g for reducing the information security risks. Each software user at the same level also defends against some of the attacks from hackers based on information sharing $K_{c}$ with sharing efficiency $\gamma_{c}$. After the security risk is effectively controlled, it takes some time to recover the information assets and reputation value, with recovery coefficient ψ.
(3) Vulnerability layer: There are two types of hackers at this level. First, type-I hackers directly attack or insert malicious software in order to obtain economic profit, beneficial information, and reputation value. Assume the number of contacts and contagions in the software supply chain be denoted as p, success rate of attack or contagion be denoted as q, increase in contagion rate be denoted as $q_{i}$, decrease in contagion rate be denoted as $q_{t}$, and the number of attacks be N. Second, type-II hackers improve their attacking technique by sharing information with type-I hackers at the same level in the hacker community. Here, the improvement in their attacking technique is denoted as $t_{h}$, the rate of attenuation of their attack technique is denoted as $t_{q}$, and the number of the type-I and type-II hackers is $\alpha_{\text{1}},\alpha_{\text{2}}$, respectively.

**Fig 3:**
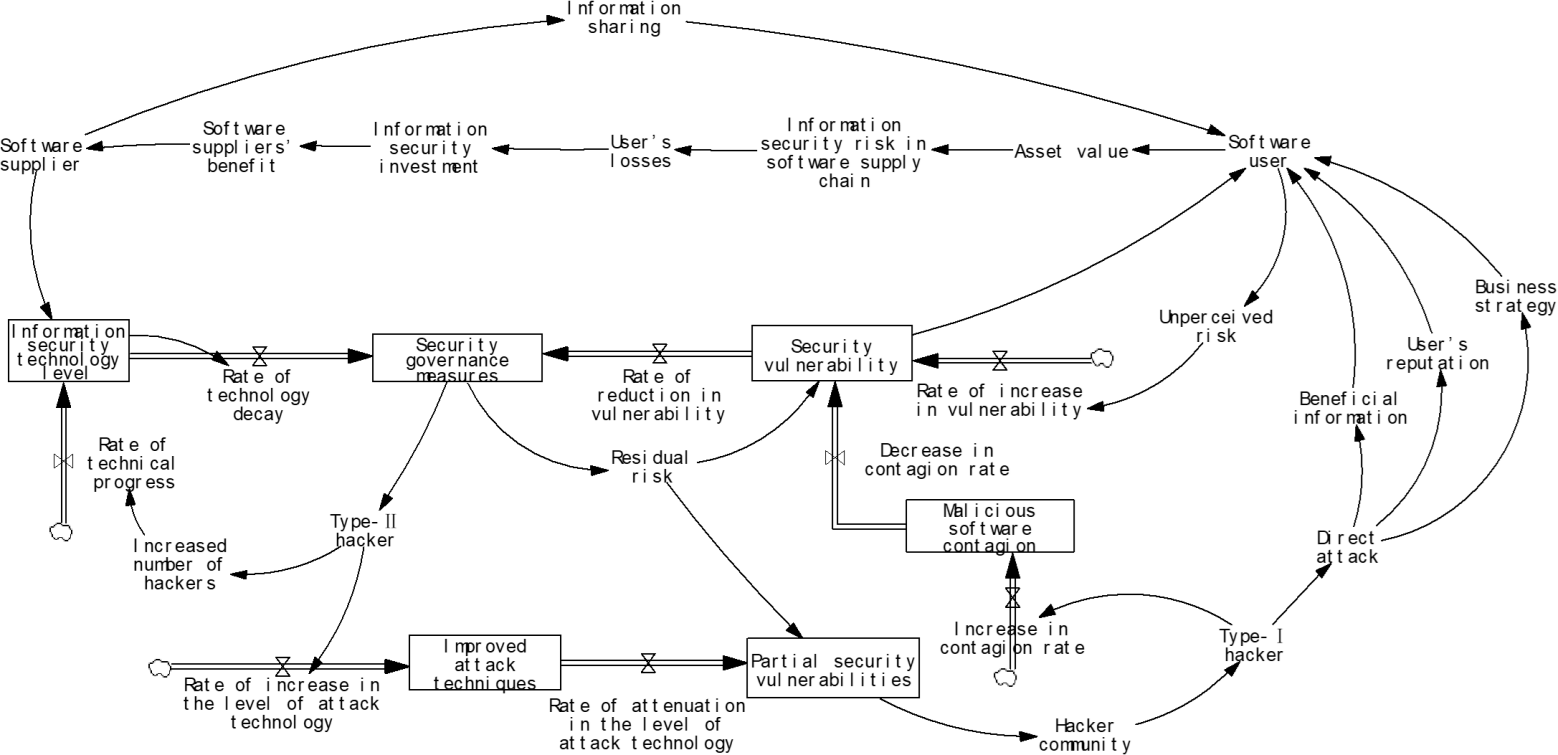
The system dynamic model based on information security risk contagion in software supply chain.

In addition, there are other factors, such as residual risk denoted as S and external information security environment factors denoted as δ. The system dynamics model is presented in Fig 3.

Considering the path of information security risk contagion in software supply chain and the relationship between various factors in the system, equations are established and parameters are set. In order to distinguish different types of variables mentioned above, flowchart is drawn to establish the functional relationships among causal variables based on the role of each participant in the software supply chain, as shown in Fig 3. The specific parameters and equations are presented in Table 1.

**Table 1:** The parameters and equations of system dynamics.

Dependent variables	Equations and parameters
type-I hacker	Number of hackers * $\alpha_{1}$
Business strategy	Direct attack * e
type-II hacker	Number of hackers * $\alpha_{2}$
Information sharing	INTEG ($K_{s}*\gamma_{s}$,1)
Level of information security technology	INTEG (($t_{s}-t_{a}$)*number of software suppliers,1)
Security governance measures	INTEG ($\left(t_{a}+V_{t}\right)*d$,1)
Software supply chain vulnerabilities	$q_{t}+V_{i}-V_{r}+S*\delta$
Malicious software contagion	INTEG ($p*\left(q_{i}-q_{t}\right)*N$,1)
Attack technology level	INTEG ($t_{h}-t_{q}$,1)
Residual risk	$\text{DELAY1}(\left[p-\left(t_{a}+V_{t}\right)*d\right]*\delta ,\text{2})$
User’s reputation	Direct attack * e
User’s losses	$I+\left(1-\frac{\text{1}}{\text{2}}*\delta *g\right)*A$
Rate of increase in vulnerability	Unperceived risk *q
Software suppliers’ benefit	$E-\left[I+\left(1-\frac{\text{1}}{\text{2}}*\delta *g\right)*A\right]$
Information security risks in software supply chain	LN(*A* + *S* + *V*_*i*_-*a*)
Software user’s benefits	$\left[\left(G+M+R\right)*K_{c}*\gamma_{c}*\psi \right]/e$
Security vulnerabilities	INTEG (S*t_*q*_)

Considering the path of information security risk contagion in software supply chain, the main causal loops are as follows:

(1) Software suppliers’ benefit → software supplier → information sharing → software user → asset value → information security risk in software supply chain → user’s loss → information security investment;
(2) Software suppliers’ benefit → software supplier → information security technology level → rate of technical decay → security governance measures → residual risk → security vulnerability → software user → asset value → information security risk in software supply chain → user’s loss → information security investment;
(3) Software suppliers’ benefit → software supplier → information security technology level → rate of technical decay → security governance measures → residual risk → part security vulnerability → hacker community → type-I hacker → direct attack → user’s reputation → software user → assets value → information security risk in software supply chain → user’s loss → information security investment;
(4) Software suppliers’ benefit → software supplier → information security technology level → rate of technical decay → security governance measures → residual risk → partial security vulnerability → hacker community → type-I hacker → direct attack → beneficial information → software user → asset value→information security risk in software supply chain → user’s loss → information security investment;
(5) Partial security vulnerability → hacker community → type-I hacker → direct attack → user’s reputation → software user → assets value → information security risk in software supply chain → user’s loss → information security investment → software suppliers’ benefit → software supplier → information security technology level → rate of technology decay → security governance measures → residual risk.

## Dynamic simulation of information security risk contagion

In view of the complexity and abstract nature of the risk of network security vulnerabilities in the software supply chain, some variables are difficult to measure and obtain. For some variables that do not affect the correctness and scientific of the simulation results due to the initial value setting, this paper assigns values based on the operability of the model and the opinions of experts in related fields. This study mainly refers to the typical software supply chain security risk cases in the “2023 China Software Supply Chain Security Analysis Report” released by Qianxin to set the relevant parameters, and collects the case materials related to the large-scale extortion attack encountered by the head management software vendor represented by Yongyou in August 2022, and improves the relevant parameter settings of the model through case interpretation. In order to avoid large fluctuations in the simulation results, the mapping method and the quantity scaling method were used to control the values at a unified level in the compilation of the system dynamics equations, meanwhile the Vensim PLE software was used for system dynamics modeling and analysis, in which month is taken as the period. The values of $\alpha_{1}$ and $\alpha_{2}$ are 0.35 and 0.65 respectively. The values of $\gamma_{s}$ and $\gamma_{c}$ are 0.57 and 0.43 respectively. d is set to 1.32.

### Simulation analysis of risk contagion process

Based on the scope of attack and the size of the loss caused in software supply chain, the attack modes in software supply chain can be divided into random attacks and selective attacks. Random attack means that the attacker randomly selects the vulnerability in a software supply chain and initiates a cyber-attack. On the other hand, selective attack means that the attacker selects a specific target group or individual to deliberately initiate a cyber-attack and cause them harm [0]. The model and the parameters presented in Table 1 are set based on the random and selective attack strategies. In this section, dynamic simulation of random and selective attacks is performed to assess their impact on software users, software suppliers, and attackers.

First, based on the multi-layer network, the rate of information security risk contagion caused by random and selective attacks is analyzed by performing simulation. Due to a larger number of type-II hackers ($H_{s}$) in the selected hacking community, the dynamic simulations are performed based on attack mode of type-II hacker. The initial values of the main parameters are presented in Tables 2 and 3.

**Table 2:** The values of parameters selected for analyzing the risk contagion process.

	$t_{s}$	$t_{a}$	u	$V_{i}$	$V_{r}$	R	e	I	g
-10%	0.42	0.05	61	0.07	0.04	48	8.53	497	3.34
+10%	0.49	0.08	68	0.09	0.08	74	19.67	878	12.86
Current 1	0.46	0.06	63	0.08	0.06	61	12.25	631	7.91
Current 2	0.45	0.05	62	0.08	0.05	63	13.26	654	8.26

**Table 3:** The values of parameters selected for analyzing the risk contagion process.

	ψ	p	$q_{i}$	$q_{t}$	N	$t_{h}$	$t_{q}$	δ
-10% −10%	1.26	377	4.67	0.29	332	4.87	0.72	0.6
+10% + 10%	1.08	626	6.36	0.83	673	3.96	1.26	0.6
Current 1	1.14	584	5.21	0.59	432	4.42	0.94	0.4
Current 2	1.21	593	5.19	0.62	457	4.51	0.96	0.4

**Fig 4:**
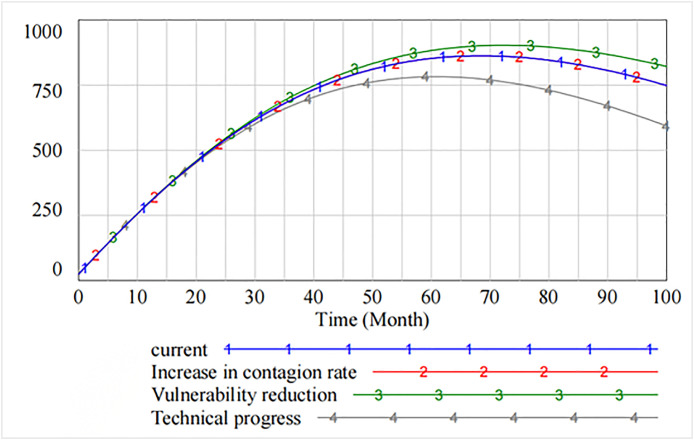
Contagion of information security risks for software suppliers under random attack.

**Fig 5:**
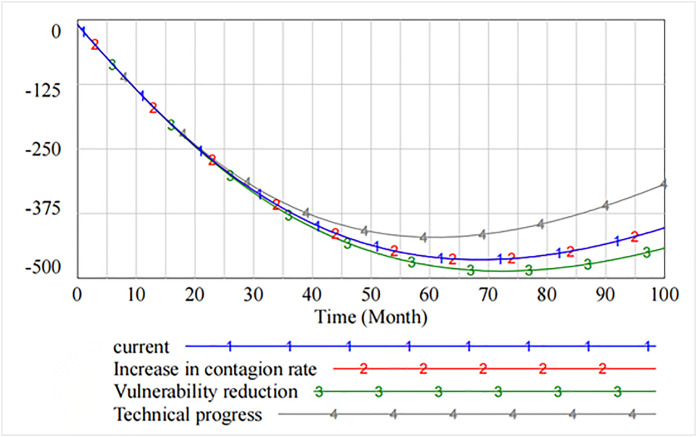
Contagion of information security risks for software users under selective attack.

Fig 4 presents the information security risks faced by software suppliers when the number of type-II hackers increases or decreases by 10% considering a random attack mode. The simulation analysis shows that the initial defense of software suppliers is not ideal when facing the cyber-attack. The effective defense only lasts for two cycles, thus leading to an increase in information security risks. At this time, an increase or decrease in the number of hackers has no significant impact on the contagion of information security risk as there is little difference between software suppliers and software users under the random attack mode. Fig 5 presents the dynamic simulation of type-II hacker initiating a selective attack mode. Under this attack, the defense of the software users is slightly different from that of the software suppliers and the risk of information security contagion increases sharply from third to 60th cycle. An increase or decrease in the number of hackers has no effect on the risk contagion. In addition, the analysis shows that from 60th cycle to the end of 100th cycle, the growth rate of information security risk contagion slows down in case of an increase in the number of hackers as compared with an increase in the number of hackers.

**Fig 6:**
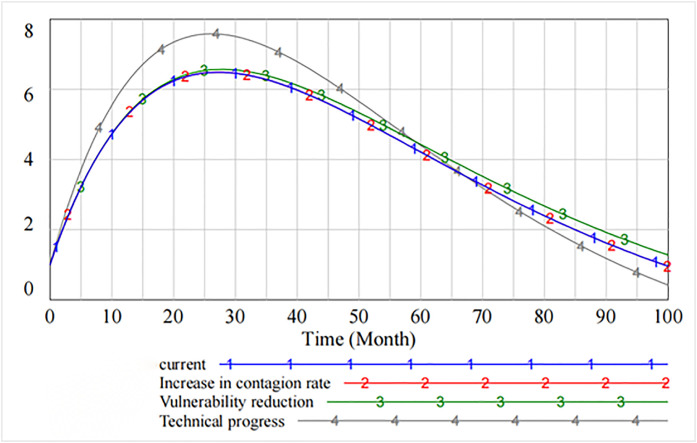
Contagion of information security risks in software supply chain under selective attack.

**Fig 7:**
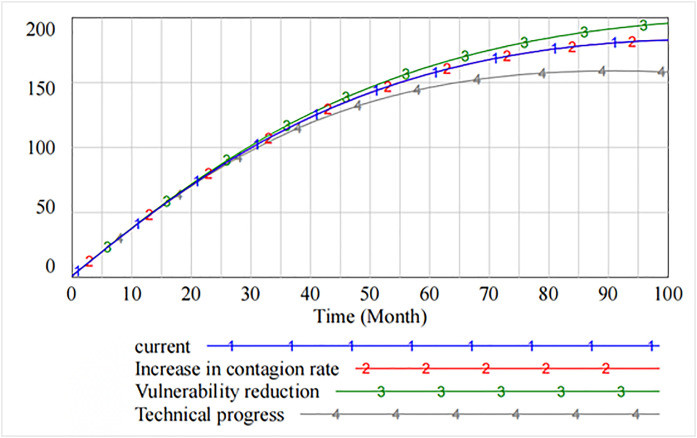
Security vulnerability in software supply chain.

Figs 4 and 5 present the information security risk contagion posed by hacker attacks from the perspective of software suppliers and software users. Fig 6 compares the effects of governance measures from the perspective of software supply chain as a whole by adjusting the threshold. The analysis shows that the initial two cycles of the software supply chain have a certain defensive effect, but at the beginning of the third cycle, the software supply chain is affected by the selective attacks. Consequently, the contagion rate of information security risks increases rapidly. At this time, the safety measures to reduce contagion rate and the rate of decrease in vulnerability have a little influence on the contagion of information security risk. The governance effect of increasing the rate of technological progress is significant after 65th cycles. Fig 7 intuitively reflects the importance of improving the rate of technological progress after 18 cycles to reduce the overall security vulnerability in the software supply chain.

In summary, the attack paths of different initial state models are different. The contagion rate of information security risk in software supply chain is affected by random or selective attack modes. The number of successful attacks varies considerably because of different cost and effort required by the hackers for initiating different attack methods. As compared to random attacks, in selective attacks, the hacker first acquires the information security vulnerability or code defect to exist in the system of a specific software user. - Consequently, these attacks have a higher success rate. After the software supply chain is attacked by the hackers, the pressure on the software suppliers multiplies in short time. However, as time goes on, the random attacks can be controlled first by gradually reducing the pressure on the software suppliers. However, when the hackers use selective attack method, the number of infected software users is relatively small due to the specificity of the attack target chosen by the hackers. Therefore, it does not force the software suppliers to pay enough attention for further developing the vulnerability patches to curb the contagion of information security risks. This is precisely because most of the targets of selective attacks are critical infrastructure, having a greater value, a wider range of impact, and greater vulnerability as well. Therefore, the selective attacks cause more damage to the software users and even the whole software supply chain systems.

### Simulation analysis of risk diffusion process

The previous section used dynamic simulation analysis for assessing the risk contagion process. This section mainly analyzes the governance of information security risks. The initial values of the main parameters are presented in Tables 4 and 5.

**Table 4:** The values of parameters selected for analyzing the governance of information security risks.

	$t_{a}$	u	$V_{i}$	R	e	I	g	ψ
Present value	0.09	56	0.09	49	7.26	428	3.24	1.34
Increase in contagion rate	0.12	62	0.11	77	20.37	657	12.85	1.52
Vulnerability reduction	0.15	63	0.13	73	20.21	665	13.51	1.41
Technical progress	0.23	65	0.12	64	18.25	632	12.91	1.38

**Table 5:** The values of parameters selected for analyzing the governance of information security risks.

	p	$q_{t}$	N	$t_{h}$	$t_{q}$	δ
Present value	377	0.43	473	3.59	0.85	0.63
Increase in contagion rate	426	0.72	586	4.56	1.46	0.62
Vulnerability reduction	453	0.69	551	4.68	1.57	0.65
Technical progress	486	0.75	569	4.41	1.94	0.68

**Fig 8:**
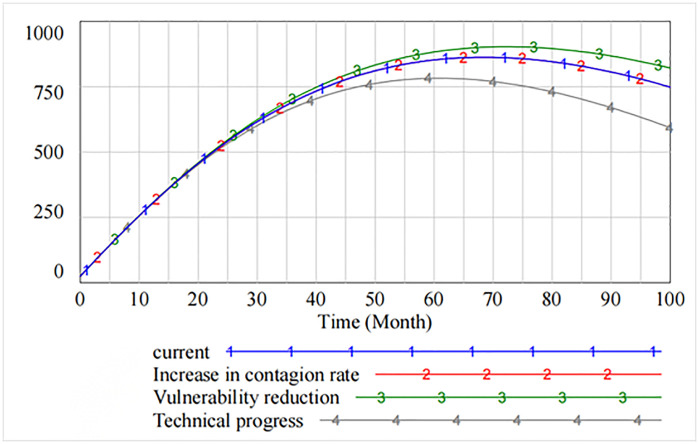
Spread of information security risks caused by partial security vulnerabilities.

Fig 8 shows that by adjusting the thresholds, some of the information security vulnerabilities first rise and then show a downward trend after peaking at 70 cycles. The analysis shows that decreasing the contagion rate does not reduce some of the resulting information security vulnerabilities. However, decreasing the rate of vulnerability increases the number of information security vulnerabilities. As compared to the original state, only increasing the rate of technical progress effectively curbs the increase in the number of vulnerabilities.

**Fig 9:**
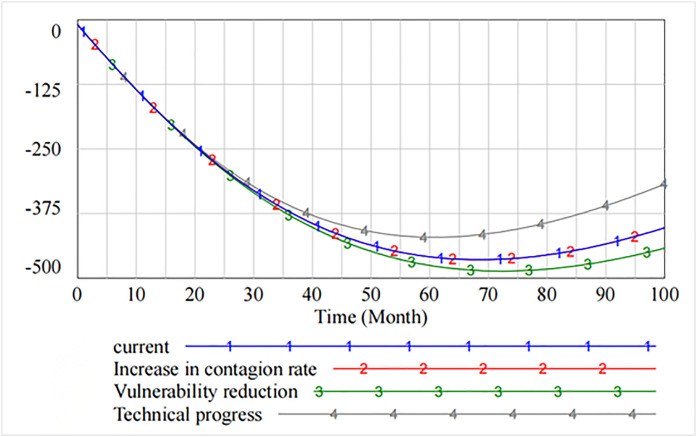
Loss of reputation of software users.

Similarly, Fig 9 shows that after software users’ reputation has been damaged up to 75 cycles, their defense level is effectively restored through three governance measures. The recovery effect increases the rate of technical progress and decreases the rate of vulnerability.

**Fig 10:**
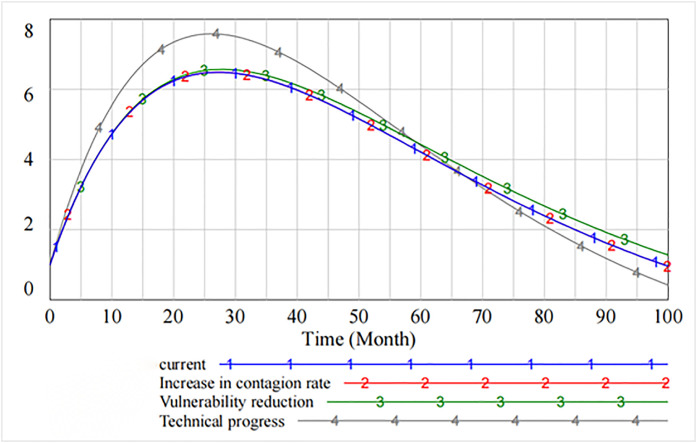
The impact of governance measures on the level of information security technology.

Fig 10 presents the impact of decreasing the contagion rate, decreasing the rate of vulnerability, and increasing technical progress on the level of information security technology. It is evident that as compared with decreasing the contagion rate and decreasing the rate of vulnerability, increasing technical progress significantly improves the level of information security, which peaks at 26th cycle and then declines. However, starting from 60th cycle, the spillover effect generated by increasing technical progress makes the level of information security lower than that of decreasing the rate of vulnerability. The marginal benefit of the rate of technical progress tends to diminish.

Therefore, as compared to decreasing the rate of vulnerability and increasing technical progress, the main module for increasing investment in information security should be to increase technical progress of software suppliers and investment in information security technology, which can effectively reduce the average response time of software suppliers when they are attacked by hackers.

### Control cross-analysis

Based on the simulation results of system dynamics, this section presents a cross-analysis of software supply chain attack and defense strategies, and analyzes the governance effects of different prevention and control strategies under different attack modes as well as the effects of different attack modes under differentiated information security prevention and control strategies.

The comparative analysis of data presented in Table 6 shows that: In terms of differentiated information security defense strategy, a combination of increasing information security investment and improving software quality level is more effective in defending against random attacks. In addition, improving the technology level of software suppliers significantly reduce the losses of software users. However, the data shows that the number of information security risk contagion, the vulnerability of the software supply chain, and the number of successful attacks by type-I and type-II hackers have not been effectively reduced. Therefore, the combined strategy of increasing information security investment and improving software quality level do not provide good defense against random attacks.

**Table 6:** The cross-analysis of attack and defense strategies in software supply chain.

Defense strategy	Increase investment in information security (SI)		Improve software quality (QI)		Improve the technology level of software suppliers (TI)	
	Random attack	Selective attacks	Random attack	Selective attacks	Random attack	Selective attacks
**SI**	702, 0.28, **151**, 5.21	884, 2.13, **377**, 12.57				
**QI**	**623**, **0.12**, 120, **5.13**	**976**, **1.28**, 279, **10.56**	635, 0.33, 122, 6.31	867, 2.41, 331, 11.43		
**TI**	678, 2.17, 114, 7.59	715, 9.43, 326, 10.79	637, 1.23, 125, 7.17	856, 2.64, 348, 11.86	**842**, 2.18, 141, 6.47	**794**, **10.59**, 352, **13.51**
**Integrated Strategy (SI + Qi + TI)**					783, 1.89, **103**, 6.51	924, 7.21, **264**, 13.08

Note: (1) The data variables in the table correspond in turn to: total risk contagion, vulnerability of software supply chain, losses of software users, and the number of successful hacker attacks, where the blackened numbers and the blackened italic numbers represent the minimum and maximum values of the variable under the attack strategy, respectively. (2) Information security investment is denoted as SI, improving software quality level is denoted as QI, and improving the technology level of the software suppliers is denoted as Ti.

When a software user arbitrarily chooses one or more information security defense strategies, the negative effects of random attacks initiated by the hackers are greatly reduced. For instance, a strategy combination of information security investment and improving software quality level can reduce the vulnerability of software supply chain under random attacks, the total amount of risk contagion and the number of successful attacks by type-I and type-II hackers. On the contrary, if the hackers adopt selective attack method, no matter whether the software user adopts single or multiple defense strategies, the vulnerability of software supply chain is greater, and its impact of information security risk contagion is far greater than that of random attacks.

### Model validity test

During simulations, the upper layer network denotes the software supplier layer and the lower layer network denotes the software user layer. Due to information overload and information security vulnerabilities, the upper layer network leads to a cascading failure of nodes representing other software suppliers. Meanwhile, the software user, as a lower layer, is unable to get the vulnerability patch from the software supplier timely because of information asymmetry, thus leading to cascading failure. At this time, the information security risk in software supply chain is transferred between the upper layer and lower layer networks, which enables the hackers in the middle layer network to use information security vulnerability for attacking the upper and lower nodes of the software supply chain. This eventually leads to the collapse of the whole software supply chain. Moreover, the model needs to undergo validity testing before being used. The proposed system dynamics model validation method not only estimates the probability of network failure or collapse in software supply chain, but also validates the simulation data for final verification of the validation model.

For initial failure nodes, selective attack or random attack is adopted. After selecting the corresponding proportion of initial failure nodes at random, the failure process of multi-layer network begins until the whole software supply chain network has no new failure nodes or the whole multi-layer network completely collapses. Then, the model simulation analysis is stopped. First, when the proportion of hacker attacks is 7%, the selective attacks are initiated on the upper and lower layers of the software supply chain, i.e., selecting vulnerable nodes with larger attack areas. In order to verify the validity of the proposed model, this section analyzes the failure scenarios of networks with 300, 500, and 800 upper and lower layer nodes respectively, i.e., the change in the failure nodes when the number of nodes in the upper and lower layer networks is equal.

**Fig 11:**
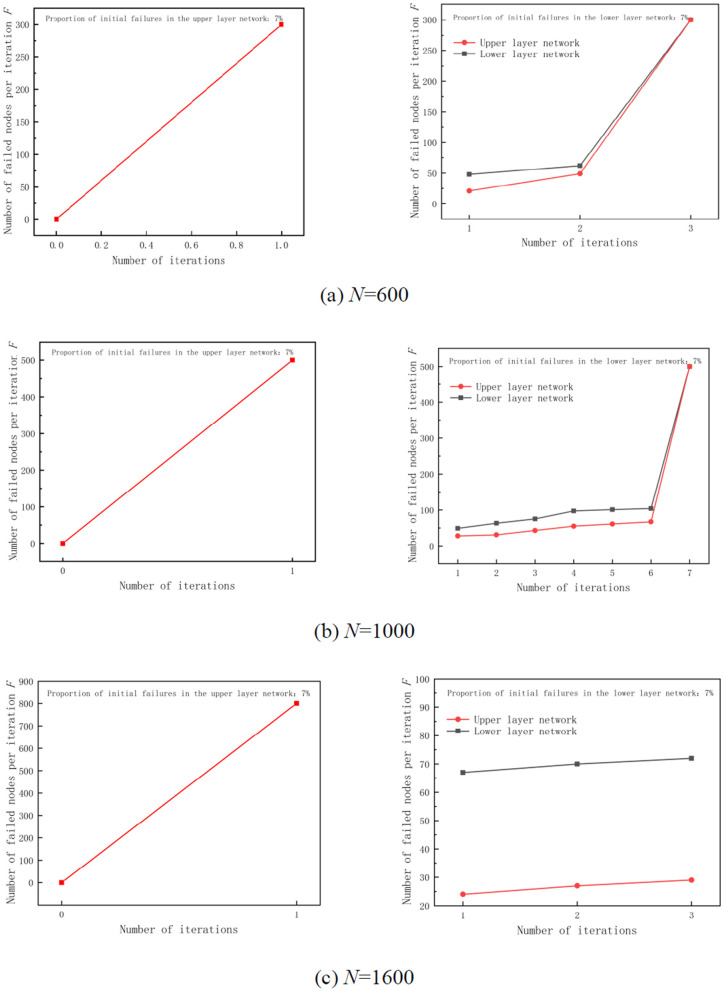
Iteration of multi-layer network failures at 7% of initial failure nodes (selective attack).

Fig 11a presents the change in the number of failed nodes per iteration when the total number of nodes in the multi-layer network is 600. Fig 11b represents the change in the number of failed nodes per iteration when the total number of nodes in the multi-layer network is 1000. Fig 11c represents the change in the number of failed nodes per iteration when the total number of nodes in the multi-layer network is 1600. As shown in Fig 11, when the failure rate of the initial selective attack nodes is 7%, regardless of whether the hacker initially chooses to attack the upper layer or lower layer network nodes, the network with 600 or 1000 nodes collapses completely. However, when the total number of network nodes is 1600, multi-layer network only crashes completely when the hacker chooses to attack the upper layer network nodes. On the contrary, if the initial attack node is chosen as the lower node, the failure process stops after three iterations and no new failure node is generated. At this point, the multi-layer network becomes relatively stable and information security risks are appropriately addressed. It is evident from simulation analysis that the scale of multi-layer network has a certain impact on the information security risk contagion.

Further analyzing the multi-layer network with 1000 nodes, it can be seen from Fig 11b that when the hacker chooses the initial attack node as the upper layer network, the whole multi-layer network will completely collapse after only one iteration. When the initial attack node is chosen as the lower layer network, whole multi-layer network completely collapses after 7 iterations. Specifically, the underload failure of the lower layer network nodes leads to the overload failure of the upper layer network. Then, the new upper layer network failure node leads to the cascading failure or even collapse of the whole upper layer network. Finally, the new upper layer network failure node leads to the failure of the lower layer network node. This iteration process is performed 7 times. After 7th iteration, the number of failed nodes is the highest. The analysis shows that the hackers attack the upper network nodes more than the lower network, when performing selective attacks, leading to a rapid failure of the software supply chain multi-layer network.

The analysis shows that when the hacker initiates a random attack with the initial attack ratio of 7%, attacking the upper layer and lower layer networks makes a little difference considering selective attack. Therefore, the initial attack ratio of 15% is proposed for random attack analysis.

**Fig 12:**
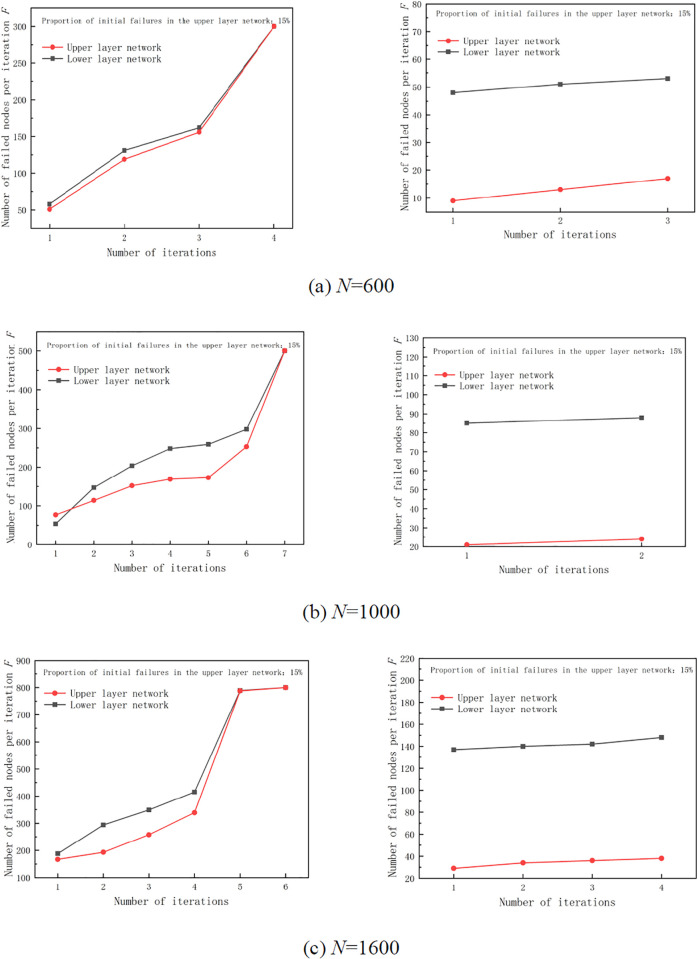
Iteration of multi-layer network failures at 15% of initial failure nodes (random attack).

When the proportion of hacker attacks is 15%, random attacks are performed on the upper layer and lower layer networks. When the number of nodes in the upper layer and lower layer networks is 500, the hacker randomly chooses 78 upper or lower nodes to attack. The analysis presented in Fig 12 shows that the multi-layer networks of three different sizes exhibit similar patterns when subjected to random attacks. When a hacker chooses the initial attack node as the upper layer network, the network eventually experiences cascading failures or crashes. When a hacker chooses the initial attack node as the lower layer network, whole multi-layer network software supply chain system automatically recovers after several iterations of failure and the multi-layer network will not experience a complete collapse.

Considering the multi-layer network comprising 1000 nodes, as shown in Fig 12b, when hacker chooses the initial attack node as the upper layer network and seven iterations are carried out, the number of failure nodes from the first iteration in the upper layer network is more than that in the lower layer network. Afterwards, with each iteration, the number of failure nodes in the lower layer network begins to increase gradually, ultimately leading to the cascading failure and collapse of the entire multi-layer network. When the initial failure node is the lower layer network, no new failure nodes are generated after two iterations. Finally, there are 24 failure nodes in the upper layer network and 86 failure nodes in the lower layer network. When hackers select an upper layer network with a random attack failure rate of 15%, it will directly cause the cascading collapse of the entire multi-layer network. However, when attacking the lower layer network, the failure of the multi-layer network will immediately stop after iterating twice. Further analysis of the above validity test shows that: (1) In cascading failure or collapse of multi-layer network software supply chain, the failure of upper layer network nodes has a deeper and wider impact on the whole multi-layer network software supply chain. (2) When the hacker chooses appropriate initial attack ratio, the multi-layer network software supply chain fails and the whole supply chain system does not collapse completely. The final number of failure nodes in the lower layer network is more than that in the upper layer network.

## Conclusions and implications

This work uses the system dynamics model to study the information security risk contagion in software supply chain with multi-layer network structure under different cyber-attack methods and the related factors that affect the information security risk contagion. We draw three principal conclusions:

When an information security risk appears in the multi-layer network software supply chain system, increasing information security investment and improving the rate of technical progress of software suppliers can reduce the rate of information security risk contagion and the overall vulnerability. In addition, to a certain extent, it reduces the damage caused to the software users. (2) Considering different defense strategies, the combination strategy of increasing information security investment and improving software quality level is more effective for the defense against random attacks. The damage caused to the software users can be significantly reduced by improving the technical level of software suppliers. (3)The negative impact of random attacks is greatly reduced when software users arbitrarily choose one or more defense strategies. In multi-layer network software supply chains, when the initial failure rate in the upper and lower layers of the network under cyber-attacks is same, and the multi-layer network structure has not yet collapsed completely, the number of failure nodes in the lower layer network is more than that in the upper layer network, i.e., the lower layer of the software user network is more vulnerable.

The key contributions of this paper are summarized as follows:(1) Novel modeling approach: We construct a multi-layer network model to analyze the contagion mechanisms of information security risks in software supply chains. (2) Micro-Level insights: The study reveals the underlying microscopic dynamics of risk contagion in this context. (3) Practical Implications: Our framework provides a new methodological perspective for assessing how information security risks compromise software supply chain integrity.

However, considering that many modern analyses of software supply chain are based on a large number of statistical data samples, the adequacy of analyzing information security risks in software supply chains from an overall conceptual model is relatively low. Therefore, future research will further explore the vulnerable network protocols in software supply chain, insecure server infrastructure, insecure coding practices, altered source code, and the system robustness and survivability of hidden malware.
